# Existence of Interhemispheric Inhibition between Foot Sections of Human Primary Motor Cortices: Evidence from Negative Blood Oxygenation-Level Dependent Signal

**DOI:** 10.3390/brainsci11081099

**Published:** 2021-08-20

**Authors:** Eiichi Naito, Tomoyo Morita, Nodoka Kimura, Minoru Asada

**Affiliations:** 1 Center for Information and Neural Networks (CiNet), Advanced ICT Research Institute, National Institute of Information and Communications Technology (NICT), 1-4 Yamadaoka, Suita, Osaka 565-0871, Japan; morita@nict.go.jp (T.M.); kimura.n@nict.go.jp (N.K.); asada@otri.osaka-u.ac.jp (M.A.); 2 Graduate School of Frontier Biosciences, Osaka University, 1-3 Yamadaoka, Suita, Osaka 565-0871, Japan; 3 Institute for Open and Transdisciplinary Research Initiatives, Osaka University, 1-1 Yamadaoka, Suita, Osaka 565-0871, Japan; 4 International Professional University of Technology in Osaka, 3-3-1 Umeda, Kita-ku, Osaka 530-0001, Japan

**Keywords:** functional magnetic resonance imaging, negative BOLD, foot movement, tendon vibration, transcallosal inhibition, cross-somatotopic inhibition

## Abstract

Interhemispheric inhibition (IHI) between the left and right primary motor cortices (M1) plays an important role when people perform an isolated unilateral limb movement. Moreover, negative blood oxygenation-level dependent signal (deactivation) obtained from the M1 ipsilateral to the limb could be a surrogate IHI marker. Studies have reported deactivation in the hand section of the ipsilateral M1 during simple unilateral hand movement. However, deactivation in the foot section during unilateral foot movement has not been reported. Therefore, IHI between the foot sections of the bilateral M1s has been considered very weak or absent. Thirty-seven healthy adults performed active control of the right foot and also passively received vibration to the tendon of the tibialis anterior muscle of the right foot, which activates the foot section of the contralateral M1, with brain activity being examined through functional magnetic resonance imaging. The vibration and active tasks significantly and non-significantly, respectively, deactivated the foot section of the ipsilateral M1, with a corresponding 86% and 60% of the participants showing decreased activity. Thus, there could be IHI between the foot sections of the bilateral M1s. Further, our findings demonstrate between-task differences and similarities in cross-somatotopic deactivation.

## 1. Introduction

Interhemispheric inhibition (IHI) between the left and right primary motor cortices (M1) plays an important role when people perform an isolated unilateral limb movement. Further, negative blood oxygenation-level dependent (BOLD) signal (termed as deactivation) obtained from the M1 ipsilateral to the limb can be a surrogate IHI marker [1]. Several studies have reported robust deactivation in the hand section of the ipsilateral M1 during simple unilateral hand movement [2,3,4,5,6,7,8]. However, there have been no reports of robust deactivation in the foot section during simple unilateral foot movement [6].

There is stronger activity in the M1 ipsilateral to a moving limb for lower- than upper-limb movement [9]; moreover, lower-limb movement shows lower lateralized activity in M1 than upper-limb movement [10]. Additionally, dynamic causal modeling using functional magnetic resonance imaging (fMRI) data has suggested no IHI effect between the foot sections of bilateral M1s during unilateral foot movement [11]. These findings suggest that IHI between the foot sections of the bilateral M1s during active unilateral foot movement is very weak or absent.

Nonetheless, IHI is important when performing bilateral out-of-phase foot movements; for example, alternating dorsi- and plantar flexion of one foot while performing plantar- and dorsiflexion of the other foot. This is because in such a case, the left or right M1 has to simultaneously control disparate actions while mutually inhibiting potential synchronized actions, likely through the transcallosal inhibitory system (cf. [12]). Therefore, IHI between the foot sections of the bilateral M1s is crucial; furthermore, the lack of deactivation in the foot section of the ipsilateral M1 (lack of functional IHI) during unilateral foot movement could result from several factors related to active foot control. In case this perspective is right, we may hypothesize that if a task could activate the foot section of the contralateral M1 passively when participants are totally relaxed, we may elucidate the deactivation in the foot section of the ipsilateral M1 using such a task.

To test this hypothesis, we employed the tendon-vibration technique. Tendon vibration of the tibialis anterior (TA) muscle activates the foot section of the contralateral M1 by most likely stimulating the muscle spindle afferent from the vibrated foot without actual movements of the relaxed foot [13,14]. In case our hypothesis is correct, this technique could allow elucidation of the deactivation in the foot section of the ipsilateral M1.

Further, we aimed to examine possible differences and similarities in the deactivation of other somatotopic sections of the M1 between active and vibration tasks given that leg movements deactivate the hand and face sections of the M1 [15]. Such cross-somatotopic inhibition is thought to suppress occurrence of an unintended movement of irrelevant body parts (e.g., hand and face) during a movement of a target body part (e.g., foot). Therefore, we assessed for between-task differences and similarities in such cross-somatotopic inhibition.

To address these points, we initially performed brain imaging analyses to determine the group effects in all participants. Here, we first identified deactivation and activation in each task. Next, we checked for brain regions showing greater deactivation in one task than in the other. Further, we checked for consistently deactivated regions during both tasks. Here, we examined brain regions within the bilateral M1s, which were defined using cytoarchitectonic maps for areas 4a and 4p [16].

Subsequently, we performed region-of-interest (ROI) analysis to examine increased and decreased activity in various somatotopic sections of the bilateral M1s during each task given the previously reported individual differences under the group deactivation effect [8]. For ROI analysis, in addition to the bilateral foot sections, we defined the trunk, hand, and face sections of the bilateral M1s using trunk, bimanual, and tongue tasks.

## 2. Materials and Methods

### 2.1. Participants

We included healthy right-handed and right-footed adults (*n* = 37; age: 37.4 ± 11.0 (mean ± standard deviation) years, range 25–59 years; 12 males). The sample size was determined using G*Power 3.1.9.7 software, which considered the use of paired *t*-test and one-sample *t*-test for ROI analysis, effect size = 0.5, α = 0.05, and power β = 0.8. This gave us 34 participants. Since our study included 37 participants, it had an adequate number of participants [17]. We confirmed participants’ handedness using the Edinburgh Handedness Inventory [18]. Moreover, we determined the dominant operating foot using a question “which leg would you use to kick a ball” in the Inventory. This question is shown to be valid to determine the dominant operating foot [19]. Among the participants, 33 and 4 participants reported that the right and both legs, respectively, were dominant. None of the participants had a history of neurological, psychiatric, or movement disorders based on self-reports. The study protocol was approved by the Ethics Committee of the National Institute of Information and Communications Technology. Before the experiments, the participants received detailed descriptions regarding the experiment and subsequently provided written informed consent. This study was conducted according to the principles and guidelines of the Declaration of Helsinki (1975).

### 2.2. fMRI Task

Before the fMRI experiment, the participants received explanations regarding the tasks to be performed in the scanner; further, they performed the tasks outside the MRI room for familiarization. Next, the participants entered the room and were placed in the MRI scanner. Their heads were immobilized using sponge cushions and adhesive tape; further, their ears were plugged. The chest, pelvis, and shin were fixed to the MRI bed using Velcro to reduce body movement during the tasks. During each task, the participants were asked to close their eyes, relax their entire body, refrain from making unnecessary movements, and only think of the assigned task.

**Figure 1:**
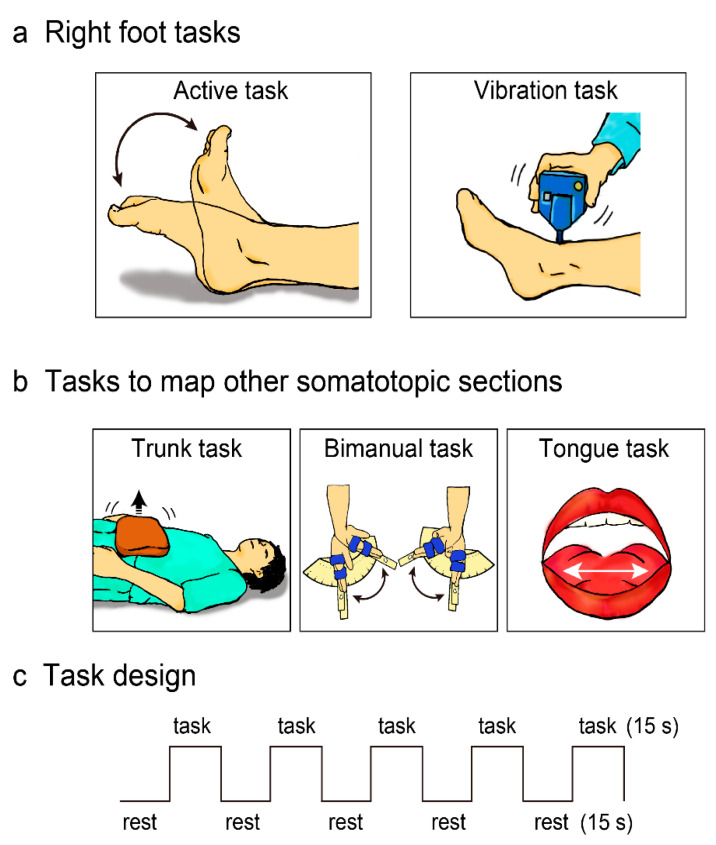
fMRI tasks and task design. (**a**): Right foot tasks. Active (left) and vibration (right) tasks. (**b**): Tasks for mapping other somatotopic sections. Trunk (left), bimanual (middle), and tongue (right) tasks. (**c**): Task design.

We prepared two foot sensory-motor tasks (Figure 1a): active dorsi- and plantar flexion of the right foot (active task) and tendon vibration of the right relaxed foot (vibration task). Additionally, to map the trunk, hand, and face sections of the bilateral M1s (Figure 1b), we prepared trunk, bimanual (hand), and tongue (face) tasks. For each task, the participants completed one experimental 160-s run, which comprised five 15-s task epochs (Figure 1c). The task epochs were separated by 15-s baseline (rest) periods. Moreover, each run included a 25-s baseline period before the start of the first epoch. During the scanning, foot, trunk, hand, and tongue movements were visually inspected by an experimenter standing beside the scanner bed.

#### 2.2.1. Active Task

Participants actively performed alternating dorsi- and plantar flexion of the right foot at 1 Hz (Figure 1a, left). They were asked to perform these movements in synchronization using a 1-Hz computer-generated tone while relaxing their left foot. We placed a supporter under the right calf and lifted the right leg off the bed to allow participants to generate these movements without their right heel touching the bed. The participants were asked to perform right foot movements within the range of their maximum dorsiflexion and plantar flexion angles, which were measured outside the scanner (average range of motion across participants was approximately 68.6° ± 15.5°). During the experimental run, the participants received auditory instructions indicating the start of a task epoch (e.g., 3, 2, 1, start) through an MR-compatible headphone. Furthermore, the participants received a computer-generated “stop” instruction that indicated the end of each epoch. Additionally, 1-Hz audio tones were generated during the rest periods. The participants heard the auditory stimuli but did not move their right foot during rest periods.

#### 2.2.2. Vibration Task

In this task, we vibrated the tendon of the TA muscle of the right foot (Figure 1a, right) during each task epoch. Similar to the active task, we placed a supporter under the right calf and lifted the right leg off the bed. Participants were asked to fully relax their feet. Details regarding the tendon-vibration technique have been previously described [13]. An experimenter (EN) continued vibrating the tendon during the 15-s task epoch; however, no stimulation was administered during the 15-s baseline period. During the task, the participants passively received this stimulation.

#### 2.2.3. Trunk Task for Mapping Trunk Sections

During this task, the participants actively pushed up a 4-kg weight placed on their abdomen (Figure 1b, left). They were asked to repeatedly perform a set of push-up and immediate relaxation in synchronization with a 0.8-Hz tone without moving their head. We used 0.8 Hz because, in our pilot experiment, some participants reported that 1 Hz was too fast, and 0.8 Hz was comfortable to follow the movements. Further, we asked the participants to keep their breathing as normal as possible during scanning. Similar to the active foot task (see above), we provided auditory instructions indicating the start of a task epoch (e.g., 3, 2, 1, start) and a “stop” instruction notifying the end of each epoch. The weight was placed on their abdomen as each task epoch started, and it was removed when the epoch was finished. Further, 0.8-Hz audio tones were generated during the rest periods where the participants heard the stimuli but did not perform movements (relaxation period).

#### 2.2.4. Bimanual Task for Mapping Hand Sections

Participants continuously exerted cyclic extension–flexion movements of their left and right wrists in synchronization with 1-Hz audio tones (Figure 1b, middle). Participants generated in-phase extension–flexion movements of both hands. We prepared a device for controlling the range of wrist motions [8]. Two stoppers were fixed onto the device to control the range of wrist motion across the task epochs and participants. They were positioned to prevent the wrist from extending beyond the straight (0°) position and flexing beyond 60°. The participants had to touch either a stopper (0° or 60°) using a hand-fixed mobile indicator in synchronization with the 1-Hz audio tones while making controlled alternating wrist extension–flexion movements. We provided auditory instructions that indicated the start of a task epoch and a “stop” instruction indicating the end of each epoch. Additionally, tones were generated during the rest periods where participants heard the stimuli but did not move their wrists (relaxation period).

#### 2.2.5. Tongue Task for Mapping Face Sections

The participants slightly opened their mouths and continuously moved their tongues to touch both cheeks in synchrony with 1-Hz audio tones (Figure 1b, right) without generating head movements [20]. Further, we provided auditory instructions indicating the start of a task epoch and a “stop’” instruction indicating the end of each epoch. Moreover, tones were generated during rest periods where the participants heard the stimuli but did not move their tongues (relaxation period).

### 2.3. MRI Data Acquisition

We acquired fMRI images using T2*-weighted gradient echo-planar imaging (EPI) sequences with a 3.0-Tesla MRI scanner (Trio Tim; Siemens, Germany) and a 32-channel array coil. We used a multiband imaging technique (multiband factor = 3), which we used in our previous study [21]. Each volume comprised 48 slices (slice thickness = 3.0 mm) acquired in an interleaved manner, which covered the entire brain. The time interval between successive acquisitions from the same slice was 1000 ms. We used an echo time of 27 ms and a flip angle of 60°. The field of view and matrix size were 192 × 192 mm and 64 × 64 pixels, respectively. The voxel dimensions were 3 × 3 × 3 mm in the x-, y-, and z-axes, respectively. For each experimental run, we collected 160 volumes. For anatomical reference, we acquired a T1-weighted magnetization-prepared rapid gradient-echo image acquired using the same scanner. The imaging parameters were as follows: TR = 1900 ms, TE = 2.48 ms, FA = 9°, FOV = 256 × 256 mm^2^, matrix size = 256 × 256 pixels, slice thickness = 1.0 mm, voxel size = 1 × 1 × 1 mm^3^, and 208 contiguous transverse slices.

### 2.4. fMRI Data Preprocessing

To eliminate the effects of unsteady magnetization during the tasks, we discarded the first 10 EPI images in each fMRI run before the start of the first epoch. We analyzed imaging data using SPM 12 (default setting: Wellcome Trust Centre for Neuroimaging, London, UK) implemented in MATLAB (Mathworks, Sherborn, MA, USA). We used a T1-weighted image as the anatomical target image. EPI images were aligned to the first image. Using this realignment procedure, we obtained head-position data that changed over time from the first frame through six parameters. All participants had a maximum displacement < 1.5 mm in the x-, y-, or z-plane and an angular rotation <0.1° about each axis during an fMRI run. Therefore, no data were excluded from the analysis. Subsequently, the realigned EPI images were co-registered to the T1-weighted structural image of each participant and spatially normalized to the standard stereotactic Montreal Neurological Institute (MNI) space [22] using the SPM12 normalization algorithm. Finally, normalized images were filtered using a Gaussian kernel with a full width at half maximum of 4 mm along the x-, y-, and z-axes.

### 2.5. Single-Subject Analysis

After preprocessing, we used a general linear model [23,24] to analyze fMRI data. In the single-subject analysis, the design matrix contained a boxcar function for the task epoch in each run, which was convolved with a canonical hemodynamic response function. To correct for residual motion-related variance after realignment, we further included the six realignment parameters in the design matrix as regressors of no interest. In the analysis, we did not perform global mean scaling to avoid inducing type II errors in the assessment of negative BOLD responses [25].

To analyze functional images, we prepared a design matrix for each participant. We generated images showing task-related deactivation (rest > task) and activation (task > rest) for each participant for each foot task. In these images, the effect of the 1-Hz sound during the active task was probably eliminated since the participants heard the sound in both the task epochs and rest periods. Further, we generated an image showing task-related activation (task > rest) in each participant for the trunk, bimanual, and tongue tasks. Individual images were used in the subsequent second-level group analyses.

### 2.6. Group Analysis for Foot Tasks

**Figure 2:**
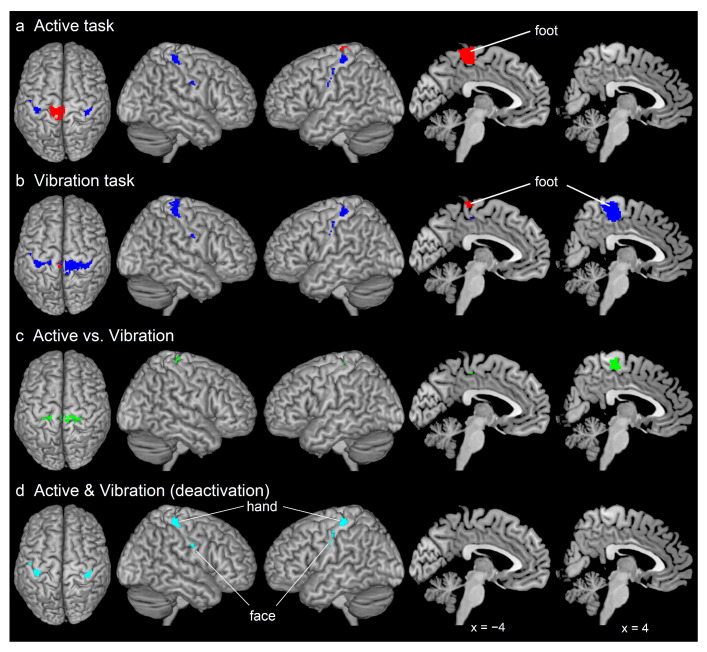
Results from brain image analyses. M1 regions showing significant activation (red) and deactivation (blue) during the active (**a**) and vibration tasks (**b**). (**c**): M1 regions are more deactivated during the vibration task than during the active task (green). (**d**): M1 regions consistently deactivated during both tasks (cyan). These activations and deactivations were superimposed on the MNI standard brain. Each row, from the left to the right, shows the top view, the right and left hemispheres, and the medial views of the contralateral (x = −4) and ipsilateral hemisphere (x = 4), respectively. Abbreviations: M1, primary motor cortex; MNI, Montreal Neurological Institute.

First, we performed second-level group analysis to examine task-related deactivation and activation in each foot task (Figure 2a,b) [26]. We conducted a one-sample *t*-test. We examined brain regions within the bilateral M1s (search space: 18,296 mm^3^), which were defined using cytoarchitectonic maps for bilateral areas 4a and 4p implemented in the SPM Anatomy toolbox [16]. Findings were reported using the family-wise error rate (FWE)-corrected extent threshold of *p* < 0.05 within the search space for a voxel-cluster image generated at the uncorrected height threshold of *p* < 0.001. The anatomical definition of the identified peaks was determined using the cytoarchitectonic map [16]. We consistently used these statistical thresholds and anatomical definitions.

Next, we examined for between-task differences in deactivation (Figure 2c) within the search space of the bilateral M1s (see above). Using a paired *t*-test, we identified brain regions showing greater deactivation in one task than in the other task among the brain regions that were deactivated in the former task. For example, when we identified brain regions that were more deactivated during the vibration task than during the active task, we applied the image of the rest > task in the vibration task as an inclusive mask (height threshold *p* < 0.001 uncorrected).

Finally, we performed conjunction analysis to examine brain regions consistently showing deactivation between both tasks within the search space of the bilateral M1s (Figure 2d) [27].

### 2.7. Region-of-Interest (ROI) Analysis of Foot Sections of M1s

**Figure 3:**
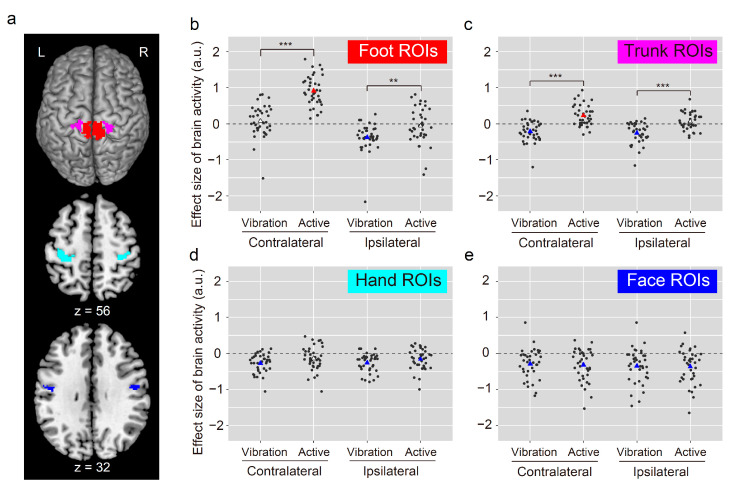
Regions of interest (ROIs) and individual brain activity within each ROI during both tasks. (**a**): The foot (red), trunk (magenta), hand (cyan), and face ROIs (blue) in bilateral M1. (**b**): We plotted individual effect sizes of brain activity in the contralateral (left) and ipsilateral (right) foot ROIs for each task. Similarly, (**c**–**e**) present data obtained from the trunk, hand, and face ROIs, respectively. Plotted points are horizontally jittered to avoid overplotting. Triangles represent the mean across participants. Red and blue triangles indicate that the mean was significantly greater or lesser than 0 (dotted line), respectively. Asterisks indicate significant between-task differences (** *p* < 0.01, *** *p* < 0.001 with Bonferroni correction). Abbreviations: L, left; R, right; a.u., arbitrary unit.

We performed ROI analysis to carefully examine the activity increase and decrease in individuals within the foot sections of the bilateral M1s during both tasks. To define the ROIs, we employed previously obtained fMRI data [13]. Here, we vibrated the tendon of the TA muscle of the left and right foot in 19 healthy adults and obtained a cluster image of brain activation around the foot section of the contralateral M1 for either foot. To define the foot section of the M1 within each hemisphere (foot ROI), we identified an overlapping region between the cluster image and the cytoarchitectonic map of areas 4a and 4p within each hemisphere (see the red sections in Figure 3a). The size of the ROIs for the contralateral and ipsilateral foot ROIs were 1544 mm^3^ and 952 mm^3^, respectively. We extracted the effect size of brain activity during both foot tasks from the contralateral (left) and ipsilateral (right) foot ROIs; further, we separately plotted data for each individual in each foot task (Figure 3b). We conducted a one-sample *t*-test for evaluating whether there was increased or decreased activity in each task compared with zero, using Bonferroni correction. Additionally, we conducted a paired *t*-test to evaluate between-task differences with Bonferroni correction (see below for the number of corrections).

### 2.8. Mapping of the Trunk, Hand, and Face Sections as Well as Their ROI Analysis

We conducted second-level group analysis to examine task-related activation (task > rest) in the trunk and bimanual (hand) tasks as well as tongue (face) tasks; further, we performed a one-sample *t*-test (height threshold, *p* < 0.001; extent threshold, *p* < 0.05, FWE-corrected). To define the trunk section of the M1 in each hemisphere, we identified an overlapping region (trunk ROI) between the cluster image and the cytoarchitectonic map of areas 4a and 4p in each hemisphere (magenta sections in Figure 3a). A similar protocol was followed for the hand (hand ROI; cyan sections in Figure 3a) and face (face ROI; blue sections in Figure 3a), respectively. The sizes of the contralateral, ipsilateral trunk, contralateral, ipsilateral hand, contralateral, ipsilateral face ROIs were 1096, 688, 3440, 2656, 576, and 624 mm^3^, respectively. We separately extracted the individual effect sizes of brain activity during both foot tasks from the trunk, hand, and face ROIs; moreover, we separately plotted the individual data in each foot task. We conducted a one-sample *t*-test to evaluate whether there was increased or decreased activity in each task compared with zero using Bonferroni correction. Given that this test was repeated 16 times (including two foot ROIs; see above), we corrected *p*-values based on the number of test repetitions (*n* = 16). Additionally, we conducted a paired *t*-test to evaluate between-task differences with the Bonferroni correction. Given that this test was repeated eight times (including two foot ROIs), we corrected *p*-values based on the number of repetitions (*n* = 8). We also provided the effect sizes (Pearson’s r values) for all the comparisons.

## 3. Results

### 3.1. Brain Deactivation and Activation during Both Foot Tasks

Consistent with our hypothesis, the vibration task deactivated the ipsilateral (right) foot section (peak MNI coordinates x, y, z = 8, −34, 72; blue section in the rightmost panel of Figure 2a); however, the active task did not deactivate this section during the active task (Figure 2a,b). Contrastingly, both tasks bilaterally deactivated the hand and face sections (Figure 2a,b). The active task broadly activated the contralateral (left) foot section (peak coordinates = −4, −24, 68; Figure 2a), with this activation extending into the trunk section (peak coordinates = −10, −40, 70). Contrastingly, the vibration task only activated a limited region of the contralateral foot section (peak coordinates = −4, −30, 68; Figure 2b). Supplementary Table S1 summarizes the results.

### 3.2. Between-Task Differences and Similarities in the Deactivation

Compared with the active task, the vibration task significantly deactivated the ipsilateral foot section (peak coordinates = 4, −24, 68) and bilateral trunk sections (peak coordinates = −18, −24, 68 for the left M1 and 14, −24, 74 for the right M1) (Figure 2c). There was no significant difference in the deactivation of any regions during the active task compared with the vibration task. Supplementary Table S2 summarizes the results. Conjunction analysis revealed that the bilateral hand and face sections (peak coordinates = −38, −24, and 60 for the left M1 hand; 38, −32, and 60 for the right M1 hand; −48, −14, and 44 for the left M1 face; 48, −10, and 34 for the right M1 face) were consistently deactivated in both foot tasks (Figure 2d). Supplementary Table S3 summarizes the results.

### 3.3. ROI Analysis

Figure 3 shows the results. All *p*-values described below were obtained after Bonferroni correction. In the contralateral (left) foot ROI (Figure 3b), there was increased activity in all and 20 participants during the active and vibration tasks, respectively. This is consistent with the finding of robust activation in the contralateral foot section during the active task (Figure 2a); however, the vibration task only activated a limited region of the contralateral foot section (Figure 2b). A one-sample *t*-test revealed significantly increased activity from 0 during the active task (df = 36, t = 13.30, *p* < 0.001, r = 0.91) but not the vibration task (df = 36, t = 1.01, *p* > 1, r = 0.17). Further, a paired *t*-test revealed significantly greater activity during the active task than during the vibration task (df = 36, t = −9.35, *p* < 0.001, r = 0.84), which indicated that the active task activated the contralateral foot ROI more than the vibration task.

In the ipsilateral (right) foot ROI (Figure 3b), 32 (86%) and 22 (about 60%) participants showed decreased activity during the vibration and active tasks, respectively, indicating that activity increased in approximately 40% of the participants during the active task. A one-sample *t*-test revealed a significant decrease during the vibration task (df = 36, t = −5.87, *p* < 0.001, r = 0.70) but not the active task (df = 36, t = −0.72, *p* > 1, r = 0.12). The paired *t*-test showed significantly greater deactivation during the vibration task than during the active task (df = 36, t = −4.06, *p* = 0.002, r = 0.56), which was consistent with findings obtained through contrast analysis of brain images (Figure 2c).

There was decreased activity in the contralateral (left) trunk ROI (Figure 3c) in 28 and 8 participants during the vibration and active task, respectively, indicating increased activity in most participants (*n* = 29) during the active task. A one-sample *t*-test revealed significantly decreased and increased activity from 0 during the vibration (df = 36, t = −4.68, *p* < 0.001, r = 0.61) and active tasks (df = 36, t = 4.70, *p* < 0.001, r = 0.62), respectively. Taken together, the active and vibration task activated and deactivated the contralateral trunk ROI, respectively, with a significant between-task difference (df = 36, t = −8.23, *p* < 0.001, r = 0.81).

There was decreased activity in the ipsilateral (right) trunk ROI (Figure 3c) in 29 and 17 participants during the vibration and active tasks, respectively, indicating increased activity in 20 participants during the active task. Therefore, the ipsilateral trunk ROI was deactivated and activated in most (78%) and approximately half (54%) of the participants during the vibration and active tasks, respectively. A one-sample *t*-test showed significantly decreased activity from 0 during the vibration (df = 36, t = −5.60, *p* < 0.001, r = 0.68) but not the active task (df = 36, t = 1.75, *p* > 1, r = 0.28). The paired *t*-test revealed significantly greater deactivation during the vibration task than during the active task (df = 36, t = −6.46, *p* < 0.001, r = 0.73). The between-task differences in the bilateral trunk ROIs were consistent with the results obtained through contrast analysis of brain images (Figure 2c).

There was decreased activity in the contralateral (left) hand ROI (Figure 3d) in most participants during both the vibration (*n* = 32) and active (*n* = 25) tasks. A one-sample *t*-test revealed significantly and non-significantly decreased activity from 0 during the vibration (df = 36, t = −6.35, *p* < 0.001, r = 0.73) and active tasks (df = 36, t = −2.51, *p* = 0.27, r = 0.39), respectively. However, there was no significant between-task differences (df = 36, t = −2.56, *p* = 0.12, r = 0.39). Taken together, the contralateral hand ROI was deactivated in both tasks.

There was decreased activity in the ipsilateral (right) hand ROI (Figure 3d) in most participants during the vibration (*n* = 33) and active (*n* = 26) tasks. A one-sample *t*-test revealed significantly decreased activity from 0 during the vibration (df = 36, t = −6.35, *p* < 0.001, r = 0.73) and active tasks (df = 36, t = −3.38, *p* = 0.03, r = 0.49), with no significant between-task differences (df = 36, t = −2.06, *p* = 0.37, r = 0.32). Therefore, the ipsilateral hand ROI was consistently deactivated in both tasks.

There was decreased activity in the contralateral (left) face ROI (Figure 3e) in most participants during both the vibration (*n* = 29) and active (*n* = 27) tasks. A one-sample *t*-test revealed significantly decreased activity from 0 during the vibration (df = 36, t = −4.23, *p* = 0.002, r = 0.58) and active tasks (df = 36, t = −4.50, *p* = 0.001, r = 0.60), with no significant between-task differences (df = 36, t = 0.38, *p* > 1. r = 0.06).

Similarly, there was decreased activity in the ipsilateral (right) face ROI (Figure 3e) in most participants during both the vibration (*n* = 30) and active (*n* = 30) tasks. A one-sample *t*-test revealed significantly decreased activity from 0 during the vibration (df = 36, t = −4.47, *p* = 0.001, r = 0.60) and active tasks (df = 36, t = −4.74, *p* < 0.001, r = 0.62), with no significant between-task differences (df = 36, t = 0.22, *p* > 1, r = 0.04). Therefore, both the contralateral and ipsilateral face ROIs were consistently deactivated in both tasks.

Finally, the deactivations in the bilateral hand and face ROIs during both foot tasks were consistent with the results obtained from the conjunction analysis (Figure 2d).

## 4. Discussion

Using the tendon-vibration technique, we elucidated the existence of deactivation in the foot section of the ipsilateral M1, indicating the existence of IHI between the foot sections of the bilateral M1s.

### 4.1. Negative BOLD Phenomenon

The physiological mechanisms underlying the task-induced negative BOLD phenomenon (deactivation) remain unclear [28], which is thought to be dependent on the stimulus type (task) and brain region [29]. However, numerous recent studies have suggested that this phenomenon may reflect neuronal inhibition [30,31,32,33,34,35,36,37,38] without exception in the central sensory-motor network [39,40].

Numerous transcranial magnetic stimulation (TMS) studies have described IHI between the hand sections of the bilateral M1s in humans [41,42,43,44,45]. However, there have been scarce studies on IHI between the foot sections of the bilateral M1s. Based on a previous finding that the negative BOLD signal (deactivation) obtained from the M1 ipsilateral to the hand during a unilateral hand movement can be a surrogate IHI marker [1], we considered deactivation in the foot section of M1 ipsilateral to the right foot as a surrogate IHI marker between the foot sections.

Physiological mechanisms underlying deactivation in somatotopic sections other than the foot sections (cross-somatotopic inhibition) within the M1 are much less unknown. However, such phenomenon has been repeatedly reported in human neuroimaging studies [5,6,8,15]. In rats, cross-somatotopic inhibition mediated by gamma-aminobutyric acid has been described from the vibrissa to the forelimb sections within the unilateral M1 [46]. A human TMS study suggested the existence of cross-somatotopic inhibition from the right face to the left hand sections between two M1s [47]. The current deactivations in the hand and face sections in both foot tasks (Figure 2d and Figure 3d,e) may suggest the existence of cross-somatotopic inhibition within and between hemispheres in humans.

### 4.2. Between-Task Differences in Foot Sections

Our findings obtained through the active task (Figure 2a) were consistent with the previously reported lack of deactivation in the foot section of the ipsilateral M1 during an isolated unilateral foot movement [6]. Contrastingly, the vibration task deactivated the foot section of the ipsilateral M1 (Figure 2b). Notably, this deactivation was consistently observed in most (86%) participants (Figure 3b). This evidence strongly indicates the existence of IHI between the foot sections of the bilateral M1s in most participants.

The main difference between active and vibration tasks is that the former requires active control of the foot while the latter requires passive kinesthetic processing of the foot [14]. Notably, the active task broadly activated the contralateral (left) foot section (Figure 2a), while the vibration task only activated the limited region of the contralateral foot section (Figure 2b). Since there was deactivation in the foot section of the ipsilateral M1 during the fully passive task (Figure 2b and Figure 3b), the lack of deactivation during active foot movement could be attributed to several factors associated with active foot control. First, foot motor control could be in bilateral mode. In daily life, we rarely move only one leg; instead, when walking, we employ alternating movements of both legs. Further, even when exercising using one leg, the other leg becomes the supporting leg. Therefore, foot motor control is basically considered as bilaterally organized. Therefore, affirmative IHI does not occur even during unilateral foot movement even in a situation where participants are lying on the bed. Second, the left leg was not fully relaxed to stabilize the posture in the MRI scanner during the right foot movement. We could not identify the specific reasons. However, it is important to indicate that a considerable percentage (about 60%) of participants showed decreased activity in the foot section of the ipsilateral M1 during the active task (Figure 3b). Therefore, the group effect of the lack of ipsilateral deactivation (Figure 2a) could be attributed to 40% of the participants showing increased ipsilateral activity rather than consistent weakness and absence of the IHI between the foot sections of bilateral M1s across participants.

### 4.3. Between-Task Differences and Similarities in the Trunk, Hand, and Face Sections

We observed that the active and vibration tasks activated and deactivated the contralateral trunk section (Figure 3c). Deactivation in the trunk section may be indicative of the existence of cross-somatotopic inhibition related to the right foot. Contrastingly, activation of this section is reflective of active recruitment during active control of the right foot, which was consistently observed in most (78%) participants (Figure 3c). This could be attributed to co-activation of the trunk muscles with the leg and foot muscles to stabilize the body posture related to right foot movement [48]. The lack of muscle activity recordings in our study impeded a clear conclusion. However, our finding that the active task activated the contralateral trunk section (Figure 3c) suggests that the brain attempted to actively recruit this section during active control of the right foot even with participants lying on the bed (Figure 2a and Figure 3c). Moreover, we observed cross-somatotopic inhibition in the ipsilateral trunk section during the vibration task across the participants; however, there was a non-significant activity increase during the active task due to individual differences (Figure 3c), similar to the findings in the ipsilateral foot ROI. Accordingly, there was robust cross-somatotopic inhibition in the ipsilateral trunk section during the vibration task. However, the activity increase in this section was not sufficiently robust as that in the contralateral section during the active task; nonetheless, there were significant between-task differences.

Finally, regarding the hand and face sections, there were no significant between-task differences in the contralateral or ipsilateral M1 (Figure 2d and Figure 3d,e). This indicates that, unlike in the trunk sections, these within- and between-hemisphere cross-somatotopic inhibitions consistently occur both during active foot control and kinesthetic processing of the foot. However, some participants may have increased activity in the contralateral hand section during active foot control.

## 5. Conclusions

Overall, the present study provides novel evidence regarding interhemispheric and cross-somatotopic inhibitions that occur during active control and kinesthetic processing of the foot. The limitation of the present study was the lack of electrophysiological evaluation of neuronal suppression underlying deactivation. Such evaluation may largely promote understanding of physiological mechanisms underlying deactivation, e.g., cross-somatotopic inhibition.

## Data Availability

The data that support the findings of this study are available on request from the corresponding author. The data are not publicly available because they contain information that can compromise the privacy of research participants.

## Supplementary Materials

The following are available online at https://www.mdpi.com/article/10.3390/brainsci11081099/s1, Table S1: M1 regions showing significant activation and deactivation during each task, Table S2: M1 regions showing significant between-task differences in deactivation, Table S3: Brain regions commonly deactivated during the tendon vibration and the active task.
