# Supplementary material for: Existence of Interhemispheric Inhibition between Foot Sections of Human Primary Motor Cortices: Evidence from Negative Blood Oxygenation-Level Dependent Signal
Source: Brain Sci. 2021 Aug 20;11(8):1099. doi: 10.3390/brainsci11081099 (PMC8393214; doi:10.3390/brainsci11081099)
Supplement: Supplementary file 1 [file brainsci-11-01099-s001.zip › brainsci-1304848-supplementary.pdf]

Supplementary materials for

Existence of interhemispheric inhibition between foot sections of human primary motor cortices: Evidence from negative blood oxygenation-level dependent signal

Eiichi Naito, Tomoyo Morita, Nodoka Kimura, and Minoru Asada

Eiichi Naito, PhD
Email: eiichi.naito@nict.go.jp

This file includes:
Tables S1 to S3

Table S1. M1 regions showing significant activation and deactivation during each task

Section	Size	Corrected p value	T-value	x	y	z
**Vibration task**						
Activation						
LH foot	24	0.03	3.99	−4	−30	68
Deactivation						
LH hand/face	479	< 0.001	6.92	−40	−24	56
			6.63	−22	−28	56
			5.96	−34	−20	46
			4.71	−40	−16	36
RH foot/hand/face	969	< 0.001	6.90	4	−30	48
			6.68	8	−34	72
			6.66	48	−10	36
			6.52	38	−32	60
**Active task**						
Activation						
LH foot	547	< 0.001	12.61	−4	−24	68
			11.38	−4	−34	72
			11.17	−10	−40	70
Deactivation						
LH hand	148	< 0.001	6.68	−40	−24	60
			5.48	−36	−22	48
RH hand	146	< 0.001	6.43	38	−32	60
			5.28	36	−22	52
			3.82	34	−36	68

RH face	79	< 0.001	5.59	48	−10	34
			5.10	54	−6	28
			4.68	40	−16	36
	80	< 0.001	5.56	−40	−14	36
			5.39	−48	−14	44
			4.20	−50	−12	36

Abbreviations: M1, primary motor cortex; LH, left hemisphere; RH, right hemisphere.

Table S2. M1 regions showing significant between-task differences in deactivation

Section	Size	Corrected p value	T-value	x	y	z
**Active vs. Vibration**						
LH trunk	62	0.001	6.93	−18	−24	68
			5.17	−22	−30	58
RH trunk	54	0.002	6.11	14	−24	74
			5.41	22	−30	64
			3.36	14	−36	70
RH trunk/foot	145	< 0.001	5.43	16	−36	58
			5.13	4	−24	68
			4.84	4	−20	60
**Vibration vs. Active**						
No significant cluster						

Abbreviations: M1, primary motor cortex; LH, left hemisphere; RH, right hemisphere.

Table S3. Brain regions commonly deactivated during the tendon vibration and the active task

Section	Size	Corrected p value	T-value	x	y	z	Anatomical identification
**Within M1**							
LH hand	148	< 0.001	6.54	−38	−24	60	
			5.48	−36	−22	48	
RH hand	144	< 0.001	6.43	38	−32	60	
			5.25	40	−28	52	
			4.53	32	−20	46	
RH face	76	< 0.001	5.59	48	−10	34	
			4.83	54	−6	28	
			4.66	40	−16	36	
LH face	73	< 0.001	5.39	−48	−14	44	
			4.71	−40	−16	36	
			4.32	−44	−10	32	
**In the whole brain**							
LH	879	< 0.001	6.84	−44	−22	58	Area 3b
			6.67	−48	−18	52	Postcentral gyrus
			5.48	−36	−22	48	Area 4p
	1509	< 0.001	5.83	−18	−80	34	Cuneus
			5.35	−38	−84	2	Area hOc4la
			5.09	−28	−84	12	Middle occipital gyrus
	96	0.004	5.04	−26	−4	−18	Amygdala
	68	0.025	4.69	−12	−80	−42	Lobule VIIa crusII
	279	< 0.001	4.52	−28	−72	−12	Area FG1
			4.15	−24	−62	−8	Lingual gyrus
			3.95	−24	−76	−6	Area hOc4v
	85	0.008	4.37	−8	−50	2	Lingual gyrus
			3.75	−8	−58	−2	Are hOc2

RH	445	< 0.001	6.47	58	−16	−10	Middle temporal gyrus
			6.29	60	−6	−10	Area TE3
			4.95	50	−16	−8	Superior temporal gyrus
	1014	< 0.001	6.43	38	−32	60	Area 4a
			6.24	42	−28	52	Area 3b
			5.59	48	−10	34	Area 4p
	2017	< 0.001	6.14	18	−80	38	Cuneus
			6.11	2	−54	50	Precuneus
			5.16	4	−42	40	MCC
	447	< 0.001	5.97	4	42	−10	Area S32
			4.76	4	54	−6	Area Fp2
	850	< 0.001	5.86	60	−48	14	Area PGa
			5.83	56	−40	8	Middle temporal gyrus
			5.59	48	−42	16	Superior temporal gyrus
	420	< 0.001	5.42	10	−78	−6	Are hOc2
			4.45	26	−68	−12	Area hOc4v
			4.30	32	−52	−16	Area FG3
	126	< 0.001	5.23	24	−12	−24	Area CA1
			5.21	30	−18	−22	Subiculum
			4.37	30	−6	−22	Amygdala
	213	< 0.001	4.97	8	−52	6	Precuneus
			4.40	16	−48	0	Lingual gyrus
	88	0.006	4.95	26	28	54	Superior frontal gyrus
			3.61	28	14	54	Middle frontal gyrus
	78	0.012	4.90	22	58	6	Area Fp1

For anatomical identification of peaks, we only considered cytoarchitectonic areas available in the anatomy toolbox with > 30% probability. The cytoarchitectonic area with the highest probability was reported for each peak. When cytoarchitectonic areas with > 30% probability were unavailable for determining a peak, we simply provided the anatomical location of the

peak. In each cluster, we reported peaks separated by > 8 mm apart from each other in order of increasing T-values. To facilitate visualization, we avoided reporting a peak for each cluster after identifying it in the same cytoarchitectonic area or in the same anatomical structure already reported for a peak with a higher T-value. For whole-brain analysis, we used a family-wise error rate cluster-corrected threshold of $p < 0.05$ using a cluster-defining threshold of $p < 0.001$. Abbreviations: M1, primary motor cortex; LH, left hemisphere; RH, right hemisphere.
